# Supplementary material for: c-JUN is a barrier in hESC to cardiomyocyte transition
Source: Life Sci Alliance. 2023 Aug 21;6(11):e202302121. doi: 10.26508/lsa.202302121 (PMC10442936; doi:10.26508/lsa.202302121)
Supplement: Supplementary file 9 [file LSA-2023-02121_TableS1.docx]

**Supplemental Table S1: Antibodies for Western blotting, immunofluorescence, and ChIP-seq**

| **Protein** | **Lot numbers** | **Dilution** | **Makers** |
| --- | --- | --- | --- |
| c-JUN | #9165 | 1:1000 | Cell Signaling Technologies, USA |
| Anti-Histone H3 | ab1791 | 1:1000 | Abcam, USA |
| Anti- H3K4me3 | ab8580 | 1:1000 | Abcam, USA |
| H3K27me3 | ab6002 | 1:1000 | Abcam, USA |
| H3K27ac | ab4729 | 1:1000 | Abcam, USA |
| TNNT2 | 565744 | 1:2000 | BD Pharmingen, USA |
| a-ACTININ | A7811 | 1:2000 | Sigma, USA |
| ISL1 | PCRP-ISL1-1A9 | 1:2000 | DSHB, USA |
| NKX2-5 | 701622 | 1:2000 | Invitrogen, USA |
